# The More Recent Methods of Treating Wounds

**Published:** 1884-02-20

**Authors:** 


### THE MORE RECENT METHODS OF TREATING WOUNDS.

When the applause which followed Professor Keith's dispassionate arraignment of Listerism before the International Congress in 1881, had died away, all eyes were directed towards Lister. But the great apostle of the carbolic spray uttered not a word. The attack, though void of impetuosity, was full of momentum and the champion was borne down. When, in course of time, he rallied sufficiently to reply, he prefaced his remarks by stating that he had never held that abdominal surgery was the touchstone of surgery, and closed them by a forced admission of the "unfortunate character" of carbolic acid. His weak defense and forced admissions struck dismay to the hearts of his followers, chiefly the Germans. The cry, "Mein Gott! Fort mit dem Spray! Lishterism is todt! Was gibts zu bleiben?" resounded throughout the hall, and the knell of Listerism was sounded.

Since that memorable day of two years ago the surgical mind has not been idle. Although Listerism, as it was represented in the carbolic spray, had received its *coup de grace*, the principle of treatment which it embodied did not suffer, and antisepticism being still recognized as an essential of treatment, the best means of securing it have engaged attention. These means, as they have thus far been published, are from German sources, the people who yielded such a slavish submission to Listerism being more zealous than any other in furthering what is really its essential part. If Lister did nothing more than to preach the gospel of cleanliness, he did this more effectually than was ever done before, and Listerism, apart from the carbolic spray, marked an era in the progress of surgery. The substances which have been proposed and are now being more or less extensively employed as substitutes for carbolic acid as applications to wounds are chiefly the subnitrate of bismuth, corrosive sublimate and iodoform. We shall endeavor to briefly describe the manner of the application of these: *

*Substitute of Bismuth Dressings*—(Kocher's Method). The application of the subnitrate of bismuth for its antiseptic properties is called the Kocher method in honor of Prof. Theodor Kocher, of Bern, Switzerland, its introducer and advocate. Kocher, by a series of experiments, proved the power of this salt both to prevent putrefaction and to prevent the development of bacteria. It is to the latter property that its virtue as a dressing is due, Kocher having shown that in the treatment of wounds, less depends upon disinfection, or the annihilation of micro-organisms, than upon antisepsis or the prevention of the development of bacteria. The desideratum of surgery is a substance which while possessing this latter property to an equal degree with carbolic acid, is devoid of the toxic properties of the latter. We have in bismuth the required substance. It is true that when employed in inordinate

*The sources of our information are chiefly, as regards the bismuth and the corrosive sublimate, editorial articles in the Annals of Anatomy and Surgery; as regards iodoform an article in the Medical Times by W. W. Jaggard, M.D.

strength it produces symptoms analogous to those of lead poisoning, but in a sufficient strength to secure antisepsis (one per cent. suspended in water) it is innocuous. Owing to its insolubility, the bismuth should be thoroughly triturated to remove all grittiness, and the emulsion thus formed should be shaken until the salt is equally diffused throughout the liquid before using. Kocher applies it in the following manner:

From an ordinary squirting bottle the wound surface is moistened at intervals in the course of an operation, so that the loose cellular tissues in particular are covered by a thin film of bismuth; at each dressing this procedure is repeated, but when the edges of the wound have been brought into apposition, bismuth, made into a thick paste, is spread upon the line of suture and allowed to dry into a crust. This method has been followed by the happiest results. The dressings of Kocher, then, consist of (1) strips of absorptive material covered by (2) a layer of gauze—both of these having been dipped into a ten per cent. solution of bismuth and the moisture thoroughly wrung out before application—and over these is laid (3) a piece of India rubber cloth, (4) cotton wadding, and (5) a dry roller bandage finishes the dressing.

To obtain the advantages which bismuth offers for securing union by first intention, certain other points must receive attention. The collection of a quantity of blood in the cavity of the wound must be prevented. This may be obtained by the forci-pressure forceps of Pean, Billroth, or by Kocher's modifications of the latter. The advantages of these forceps consist in the fact that they grasp firmly when applied, take up a limited amount of space, and are absolutely aseptic. Hemorrhage, therefore, can be promptly controlled. Since extravasation from blood and lymph vessels cannot be absolutely prevented by ligature, a uniform compression of the edges and surface of the wound throughout its whole extent is necessary. This may be obtained by the use of Martin's rubber bandage. Kocher uses a combination of adhesive plaster with strips of rubber stitched or pasted together; in order to secure permanent adhesion between the rubber and the plaster they should be subjected to powerful pressure. A strip of rubber, with adhesive plaster strips upon either end, if this method is used, should be prepared for an expected operation. If this compress dressing can be applied with accuracy the surgeon will be justified, hemorrhage having been controlled and bismuth having been applied, in closing the wound immediately after an operation.

By means of the rubber and adhesive plaster strips pressure may be secured sufficient to prevent extravasation without materially impeding circulation. The most prominent feature in this dressing is the absence of encircling bandages, since by the use of good adhesive plaster the rubber strips can be tightened or relaxed to suit all circumstances.

With the rapid healing of wounds following the use of these methods, care must be taken against too early exertions upon the part of patients, subjecting them to the possible detachment of emboli from imperfectly organized thrombi in the several vessels. Experience shows that there is particular danger of this in wounds of the neck and other parts where the ligature of a large vein may have been necessary.

The perfect asepsis secured by bismuth is the chief point in its favor. For instance, in a case of knee-joint disease with fungous degeneration, where the joint was opened and the diseased tissue removed, then dressed with bismuth and the secondary suture, without the use of drainage tubes, rapid and uncomplicated recovery ensued.

Another advantage of bismuth, if used according to this method, is the entire absence of direct systematic effects. The great simplicity of the method, and the absence of cumbersome details and apparatus is of great advantage to the surgeon. The convenience and freedom from annoyance to the patient as well, is greatly in its favor. The application of the salt upon a fresh wound surface causes, momentarily, a smart, burning sensation. On the second day, when the secondary suture is applied, the patient no longer complains when the bismuth irrigation is used.

As an antiseptic, bismuth is of greater use than iodoform on account of its insolubility. If it is desired to disinfect hands or instruments, or if an infected wound and the integument surrounding it must be disinfected, *i. e.*, if pathogenic organisms, which have found entrance to the wound, are to be destroyed, soluble antiseptics, like carbolic acid or corrosive sublimate, should be used.

*Corrosive Sublimate Dressings*—(Kummell's method.)—The credit of using the mercuric bichloride both for the purposes of irrigation and wound dressings, belongs to Kummell, of Hamburg, whose attention was called to the subject through the labors of Koch Experiments have shown that in solutions of this salt, of strength of from 1:1,000 to 1:20,000, bacteria are killed and their further development checked. It is the most powerful of antiseptics, as shown by the experiments of Koch and Pasteur on the bacillus of gangrene of the spleen. This bacillus resisted all other antiseptics, but was readily destroyed by a solution of corrosive sublimate, 1:1,000.

Acting upon the possibilities which these experiments suggested, Kummell proceeded to make a practical test of the applicability of the mercuric bichloride as an antiseptic wound dressing. Disappointed at the comparatively slight antiseptic effect of the five per cent. carbolic solution in general use for purposes of irrigation, he at first used a 1 to 5,000 solution of mercuric bichloride for the same purpose in Schede's wards in the Hamburg General Hospital. He gradually increased the strength of the irrigating fluid to 1 to 1,000, and even to a one per cent. solution, without the slightest trace of dangerous symptoms supervening. The five per cent. solution of carbolic acid was used for the spray and as a bath for the instruments. The latter, if exposed to the action of the mercuric bichloride for only a few seconds, will become blackened by the rapid amalgamation upon their bright surfaces ; nickel-plating will not protect them from this injury. Enveloped as the heads of the operator and his assistants are by the spray, a sufficient amount of the latter, were it to be even an atomization of a very dilute solution of the mercuric bichloride, might be productive of troublesome if not dangerous symptoms. It is evident, therefore, that for neither of the above purposes can the corrosive sublimate solutions be made available. In two patients treated with the one-per-cent. solution the constitutional effects of the drug appeared ; in one salivation taking place, and in the other a diarrhœa, which latter was afterwards thought to be of a tuberculous origin. Recovery took place in both cases in a few days without the necessity of a removal of the dressings. Both of these patients had suffered previously from iodoform intoxication, suggesting a peculiar susceptibility to the action of antiseptics.

Since the introduction of the bichloride solution as an irrigating fluid, Kummell deprecates the use of sponges, except when absolutely required in the operation itself ; the cheapness of the sublimate justifies its free use in cleansing the parts. The floor and the walls of the operating-room are scoured and scrubbed with the solution, and no accident has yet occurred to either attendants or patients. After seven months' use of the substance in this free manner, he avers that, with the exception of the two cases above alluded to, no ill effects whatever have been attributed to it.

In a person with an extremely sensitive skin the dressings of mercuric bichloride may, according to Kummell, produce an eczematous condition of the parts surrounding the wound. This does not occur from a simple irrigation of the wound and the surrounding tissues ; but when it does take place, which is rare, it arises from the constant contact of the wound and its neighborhood with dressings impregnated with the sublimate. In wounds in which putrefaction changes have already occurred, the mercuric bichloride solution quickly banishes the odor and arrests the septic processes.

The dressings devised by Kummell consist of sublimated gauze and cotton, sublimated silk, sublimated cat-gut, sublimated oil, and sublimated inorganic dressing materials. These latter comprise powdered glass, sand, coal ashes, asbestos, lint made from spun glass, and, for purposes of drainage, capillary threads of spun glass.

Sublimated gauze and cotton are designed to take the place of carbolized gauze and cotton. They are made hygroscopic in the usual manner (benzine washings), and then impregnated with the following :

| | |
|---|---|
| Corrosive sublimate, | 10 parts, |
| Alcohol, | 4,490 " |
| Glycerine, | 500 " |

The moisture is pressed out with a clothes-wringer. If a stronger solution of the corrosive sublimate is used, eczema and bullæ of the integument will be produced.

For sutures, sublimated silk is used. It is previously prepared as for carbolized silk, by Hegar's method, and then boiled for two hours in a one per cent. solution of corrosive sublimate ; it is then transferred to a one-tenth of one per cent. solution of the same, where it is kept ready for use.

For ligatures, sublimated cat-gut is used. The unprepared catgut is first immersed in a one per cent. solution of the corrosive sublimate for twelve hours, and then firmly wound upon spools, and preserved in an alcoholic solution of half of the above strength, to which is added ten per cent. of glycerine. Cat-gut thus prepared is very flexible, and it is asserted by Kummell will give perfect immunity against infection, whatever might have been the condition of the gut prior to its preparation.

Sublimated oil, of the strength of one per cent. is employed for uniformity's sake. This oil, however, as shown by Wolfhugel and Knorre, of the Imperial German department of health, possesses no more powerful antiseptics than carbolized oil.

In order to avail himself of the properties of the mercuric bichloride in the form of a powder dressing, Kummell experimented with a variety of substances, and finally selected common white sand. This is first well sifted and then heated in a covered vessel over a coal fire. On cooling, it is mixed with an ethereal solution of mercuric bichloride and kept for use in a glass-stoppered bottle. The solution is made by dissolving 10 grammes (ʒijss) in 100 grammes (℥iij) of ether. This quantity is sufficient to perfectly impregnate 10 kilogrammes (22 pounds avoirdupois) of previously heated sand.

The sublimated sand can be used in many ways as a wound dressing. It may be used as any other powder dressing in filling in previously disinfected and bloodless wound cavities where primary union cannot be hoped for. In such a case the dressing should not be covered with an impermeable covering, such as parchment paper, gutta-percha tissue or Mackintosh, for the reason that it is desirable not to prevent drying of the secretions in the sand, healing taking place as under the scab. The under dressing is not to be disturbed for several weeks, or until healing has taken place. The outer dressing of sublimated gauze is removed from time to time as the secretions find their way through and dry thereon, and new pieces are applied in their stead. When irrigation of the parts is required, the solution of mercuric bichloride is used, and new sand applied where the old has been washed away. Wounds thus treated were always found to be aseptic and odorless. The outer pieces of gauze in which the secretions had collected were, however, sometimes found to possess a glue-like odor.

The sublimated sand tends to keep the wound dry by absorbing whatever secretions occur, and likewise by decreasing their amount. The discharge is often very limited indeed; large cavities, such as are left after excision of a joint, being filled with the sublimated sand, are kept so for a week without the appearance of a single drop of pus. The rapidly forming granulations push the sand outwards from the bottom of the cavity, and, upon being finally removed, disclose the wound firmly cicatrized as under the scab. No fear need be entertained of the sand being imprisoned in the granulations and remaining in the healed wound; should this occur, which is very rare, the particles of sand in the tissues will be found to be perfectly harmless. A great advantage of the sublimated sand dressing will be found in its applicability to compound fractures and other injuries requiring the use of a plaster of Paris dressing. Here, on account of the scantiness of the discharge under this dressing, the fixed apparatus may be left in situ for several weeks without being disturbed.

By means of an insufflator the fine sublimated sand may be instilled into sinuses, various fissures and deep recesses.

The sublimated sand will be found equally convenient of application in the case of wounds in which primary union is expected to occur. After the wound is brought together and drainage threads of spun glass placed in position, a layer of lint, made of spun glass, and disinfected in the solution of the strength of 1 to 1,000, is to be laid along the line of incision to prevent the sand from coming in contact with and between the edges of the wound. The sand may now be placed on in sufficient quantities and covered by several layers of sublimated gauze. This method is susceptible of general application to amputation and resection wounds, when very firm compression is not needful, and where the bone underlying the tissues affords a firm support.

A rise of temperature occurring within the first three days, the so-called aseptic fever of Lister, and which is of common occurrence when other antiseptics are used, was entirely absent when the above form of dressing was employed, according to its originator.

In cases in which primary union, or union by first intention is sought, the grains of sand (when this is applied) might lead to failure. This fact caused Kummell to devise bags made of coarsely textured cotton cloth, well disinfected and rendered hygroscopic, and filled with the antiseptic sand. The sand-bags, however, proved too heavy and cumbersome, and to overcome these objections he substituted finely sifted coal ashes for the sand. Cushions thus made are light, soft and pliable. Before applying it the bag is moistened, in order to facilitate absorption of the discharge.

Another feature of Kummell's antiseptic method consists in the employment of glass wool or wadding. This material is derived from annealed glass rods, which are capable, when heated, of being drawn into long delicate fibres having a diameter of from 0.01 mm. to 0.006 mm. They have a snowy white appearance, with a silk-like lustre, and are very flexible. They can be braided together into bunches, or compressed into masses for convenience of use. Bunches of these can be tied together, and make most efficient capillary drains. The strands are so soft that there is no danger of broken particles entering the tissues. This glass wool can be thoroughly purified by chemical agents, and after washing, can be rendered antiseptic by immersing it in the ethereal solution of mercuric bichloride used in the preparation of the sublimated sand. A dry dressing thus prepared will absorb secretions very rapidly. A much simpler and more effectually antiseptic dressing of this material consists of masses of it, disinfected by submersion in concentrated sulphuric acid, and preserved in a one per cent. solution of the mercuric bichloride ; when needed this is squeezed out of the solution and applied moist in thin layers directly to the wound. Where the sand or ash cushions are used, a thin layer of this glass wool will serve as a protection to the wound without detracting from the absorbent powers of the rest of the dressing.

*Iodoform Dressings*, (Billroth's).—Billroth is scrupulously attentive to the details of antiseptics, insisting on the thorough disinfection of the apartment in which the operation is made, the hands of the operator and his assistants, and of the knives and other instruments employed. A two-and-a-half per cent. solution of carbolic acid is what he employs for the disinfection of persons and instruments. After completion of the operation the wound is irrigated with a solution of the same strength and thorougly dried to remove all blood-coagula or foreign water. Great care is also bestowed on the drainage of wounds, to secure which drainage tubes are freely employed if necessary.

Billroth's dressing of the wound differs from Lister's chiefly in the substitution of carbolized gauze by iodoform, in the form of powder or of gauze.

Outside of the operating-room, iodoform is employed:

(1). As a powder, sprinkled over wounds, as upon the perineum, by means of Dr. Wolfler's iodoform-duster.

(2). As gauze, which may be either (*a*) dry, "hydrophile," or (*b*) adhesive. For the preparation of hydrophile iodoform gauze (*a*), a coarse, unbleached muslin, which has been deprived of its fatty particles, is placed in a basin, washed with carbolic acid, and is sprinkled with iodoform in the form of powder until the cloth assumes a yellow color. According to the thoroughness of this operation, the gauze contains from ten to twenty per cent. of iodoform. Fifty grammes of iodoform are sufficient to impregnate six and a half metres of muslin. Hydrophile gauze costs in Vienna about eight cents per metre.

For the preparation of the adhesive iodoform gauze (*b*), the muslin, deprived of its fatty particles, is saturated with a mixture of alcoholic solution of colophonium and glycerin. The gauze is dried carefully and impregnated with iodoform in the same manner as the hydrophile. For six metres of gauze it requires two hundred and thirty grammes of iodoform and one hundred grammes of colophonium, which is dissolved in twelve hundred grammes of ninety-five per cent. of alcohol, to which fifty grammes of glycerin are added. This adhesive iodoform gauze costs in Vienna about thirty-two cents per metre.

The chief function of adhesive iodoform gauze is the arrest of hemorrhage in cases of parenchymatous hemorrhage.

(3). As iodoform glycerin. This preparation consists of ten to twenty parts of iodoform to one hundred parts of glycerin, and is employed for injection into cold abscesses after the evacuation of pus by puncture or incision.

(4). As iodoform collodium. This preparation is composed of iodoform one part to collodium ten parts, and is used in enormous quantities in the ambulatorium. It is a sovereign remedy for cuts and slight bruises. An ethereal solution of iodoform (one part iodoform, seven parts ether) forms a very convenient covering for syphilitic scleroses, and for mucous patches in the buccal cavity.

(5). As iodoform bacilli. The formula for this preparation, as it is found in the pharmacy of the General Hospital, is :

| | | |
|---|---|---|
| ℞ Iodoform, pulv | | 20.0 |
| Gummi arabicæ | } | aa 2. |
| Glycerini | } | |
| Amyli | } | |

Fiant bacilli diversi magnitud.

The value of these bacilli cannot be over-estimated when fistulous tracts or inaccessible wound surfaces are to be medicated. In endometritis, cystitis, pyothorax, and certain urethral affections, the iodoform-bacilli are evidently of great worth.

(6). As iodoform-vaseline. This salve varies in the amount of the drug contained from twenty to fifty per cent., and is used as an application to venereal ulcers.—*Medical Age.*

